# The Qualitative Scoring MMSE Pentagon Test (QSPT): A New Method for Differentiating Dementia with Lewy Body from Alzheimer’s Disease

**DOI:** 10.3233/BEN-120319

**Published:** 2013

**Authors:** Paolo Caffarra, Simona Gardini, Francesca Dieci, Sandra Copelli, Laura Maset, Letizia Concari, Elisabetta Farina, Enzo Grossi

**Affiliations:** ^1^Department of NeuroscienceUniversity of ParmaParmaItaly; ^2^Outpatient Clinic for the Diagnosis and Therapy of Cognitive DisordersAUSLParmaItaly; ^3^Fondazione Don GnocchiMilanIRCCSItaly; ^4^Medical Department Bracco SPAMilanItaly

**Keywords:** Dementia with Lewy Body, Alzheimer's disease, copy of pentagons, differential diagnosis, MMSE

## Abstract

The differential diagnosis across different variants of degenerative diseases is sometimes controversial. This study aimed to validate a qualitative scoring method for the pentagons copy test (QSPT) of Mini-Mental State Examination (MMSE) based on the assessment of different parameters of the pentagons drawing, such as number of angles, distance/intersection, closure/opening, rotation, closing-in, and to verify its efficacy to differentiate dementia with Lewy Body (DLB) from Alzheimer's disease (AD). We established the reliability of the qualitative scoring method through the inter-raters and intra-subjects analysis. QSPT was then applied to forty-six AD and forty-six DLB patients, using two phases statistical approach, standard and artificial neural network respectively. DLB patients had significant lower total score in the copy of pentagons and number of angles, distance/intersection, closure/opening, rotation compared to AD. However the logistic regression did not allow to establish any suitable modeling, whereas using Auto-Contractive Map (Auto-CM) the DLB was more strongly associated with low scores in some qualitative parameters of pentagon copying, i.e. number of angles and opening/closure and, for the remaining subitems of the MMSE, in naming, repetition and written comprehension, and for demographic variables of gender (male) and education (6–13 years). Twist system modeling showed that the QSPT had a good sensitivity (70.29%) and specificity (78.67%) (ROC-AUC 0.74). The proposed qualitative method of assessment of pentagons copying used in combination with non-linear analysis, showed to be consistent and effective in the differential diagnosis between Lewy Body and Alzheimer’s dementia.

## 1. Introduction

Dementia with Lewy Body (DLB) is the second most frequent variant of degenerative dementia in elderly people, after Alzheimer's dementia (AD) [1], accounting for 10–15% of cases at autopsy [2]. Often DLB is mistaken for AD, because of their overlapping features [3,4].

DLB is characterized by the presence of cognitive, psychiatric and motor symptoms [2,5], including progressive cognitive decline with fluctuating course sometimes associated with recurrent episodes of confusion, extrapyramidal signs in 25–50% of the cases, mostly rigidity and bradykinesia [6]. Patients usually show a combination of cortical and subcortical neu-

ISSN 0953-4180/13/$27.50 © 2013 – IOS Press and the authors. All rights reserved ropsychological deficits [7,8] with attentional, executive and visuospatial impairments. Fluctuating cognition especially in attention and alertness and visual hallucinations are also reported as core features.

Common neuropathological features include Lewy bodies, clumps of alpha-synuclein and ubiquitin protein located in limbic cortex and neocortex, subcortical and brainstem nuclei, particularly the substantia nigra, as well as in basal forebrain and locus coeruleus, but less frequently in the occipital cortex [9].

Lewy bodies are commonly found also in a similar proportion of patients with AD as well as in normal subjects. Otherwise, most cases with both brainstem and neocortical LBs are often characterized by some degree of AD pathology. This includes diffuse, neuritic plaques and modest medio-temporal neurofibrillary tangles (NFTs), suggesting sufficient features to meet accepted neuropathological criteria for AD [9, 10].

Thus similar neuropathological features might explain the difficulty to clinically differentiate DLB from AD highlighting at the same time that the proper identification is of primary importance for a correct therapeutic approach.

Many studies compared the neuropsychological features of DLB patients with those of AD [11–13], resulting DLB patients more severely affected on visuoperceptive, executive and attentional functions than AD, being commonly more impaired on memory [14].

Stavitsky et al. [12] found more significant differences between the two syndromes in the earlier stages of dementia. The presence of extrapyramidal signs, psychiatric symptoms, and constructional deficits, is strongly suggestive for DLB, whereas recognition memory impairment is more likely to be similar in the two groups and seems to differentiate them in later stages, being progressively more rapid and severe in AD patients. Palmqvist et al. [4] suggested the importance of identifying tests able to easily recognize DLB *ab initio* and one of the item included in the Mini-Mental State Examination (MMSE) [15], such as the copy of pentagons, was found to be a good target. Using a qualitative analysis approach of the pentagons copy, Ala et al. [16] were able to differentiate autopsy proven subjects with DLB from AD, reaching a sensitivity of 88% associated however with low specificity of 59%. Comarck et al. [17], referring to the pentagon copying assessment scale [18], suggested that the pentagon copy correlates to global cognitive impairment in AD, whereas in DLB it is more influenced by selective visual perception and apraxia.

Despite the wide use of MMSE to assess general cognitive abilities, qualitative and reproducible methods to evaluate the pentagon sub-item are lacking.

The aim of the present study was firstly directed to introduce a new qualitative scoring method for the pentagons copy test (QSPT) and to ascertain the inter-rater and intra-subjects reliability. Secondly we applied the QSPT to patients with DLB and AD in order to verify the potential role of this new scoring method in differential diagnosis between these two variants of dementia, by comparing linear and non-linear statistical analysis, such as the artificial neural networks.

## 2. Materials and methods

Two trained neuropsychologists (SC and FD) unaware of the diagnosis, independently scored the copy of pentagons test produced by forty-six AD patients and forty-six DLB patients using the QSPT, in order to measure the inter-rater reliability.

To assess the intra-subjects reliability of QSPT we compared the performance of a group of eleven patients randomly chosen from the sample served as intra-subjects being evaluated at baseline and after two weeks.

### *2.1. Participants*

Forty-six consecutive patients meeting NINCDS-ADRDA criteria [19] for the diagnosis of AD and forty-six patients meeting clinical criteria for the diagnosis of DLB [5] participated to the study. AD and DLB subjects underwent to an extensive neuropsychological battery which included assessment of memory, attention, executive and visuo-spatial abilities, language and neuropsychiatric profile [20–23]. The pharmacological treatment was administered not to all the patients, due to previous side effects or contraindications in AD and DLB groups. Among AD patients only 60% received anticholinesterase treatment, which was also present in only 65% of DLB individuals. 32% of DLB patients received antiparkinsonian drugs, while less than 20% and 25% had antipsychotic and antidepressant respectively. DLB patients were randomly recruited by a nurse blind to the study from the database of 102 patients who took part to the previously published study [24].

The two groups did not differ for age (mean AD 78.24 $\pm$ 5.18; mean DLB 77.87 $\pm$ 5.78), education (mean AD 6.76 $\pm$ 3.6; mean DLB 7.46 $\pm$ 3.99),

Table 1
Qualitative scoring method for the pentagon copying test (QSPT)

| Parameters | Performance scores | Assigned scores |
|---|---|---|
| 1. Numbers of angles | 10 | 4 |
| | 10 ± 1 | 3 |
| | 10 ± 2 | 2 |
| | 7–5 | 1 |
| | < 5 or > 13 | 0 |
| 2. Distance/Intersection | Correct Intersection | 4 |
| | Wrong Intersection | 3 |
| | Contact without Intersection | 2 |
| | No contact, distance < 1 cm | 1 |
| | No contact, distance > 1 cm | 0 |
| 3. Closure/opening* | Closing both figures | 2 |
| | Closing only one figure | 1 |
| | Opening both figures | 0 |
| 4. Rotation** | Correct orientation of both figures | 2 |
| | Rotation of one figure (either one figure is absent or it is not a pentagon then it is not assessable) | 1 |
| | Rotation of both figures (or both not assessable like pentagons) | 0 |
| 5. Closing-in | Absent | 1 |
| | Present | 0 |
| Total | Sum of 1+2+3+4+5 | 0–13 |

*Figure is considered close even though two sides do not touch each others but the distance is ⩽ 1 mm.
**When there is not a figure or figure is not a pentagon (then rotation is not assessable) score is 0. When rotation is less than 45°, figure is not considered rotated. Tremor is ignored.

MMSE score (mean AD 19.37 ± 4.5; mean DLB 19.3 ± 4.46) and disease duration (mean AD 3.5 ± 1.6 year; mean DLB 3.7 ± 1.8 year).

Eleven patients suffering from AD served as intra-subject evaluation (see procedure).

Procedures are done in accord with the ethical standards of the Committee on Human Experimentation of the institution in which the experiments were done in accord with the Helsinki Declaration of 1975. This research protocol has been approved by the local Institutional Ethical Committee.

### 2.2. Procedure

The new scoring method included five criteria of judgment as follows: 1) numbers of angles; 2) distance/intersection between the two figures; 3) closing/opening of the contour; 4) rotation of one or both pentagons; 5) closing-in and a total score corresponding to the sum of individual scores of each parameter, ranging from 0 to 13.

For each parameter an arbitrary score was assigned where zero indicated the worst performance (see Table 1 for detailed description). Tremor was ignored. When participants executed more than one copy of pentagons, the best copy was graded.

Two trained neuropsychologists (SC, FD) blind to the clinical diagnosis evaluated independently the pentagons drawings. Eleven patients (mean age 77.8 ± 5.60, mean education 6.95 ± 3.7), serving as intra-subject sample, were asked to perform the copy of pentagons at baseline and after two weeks interval.

As a further step we were interested to explore the potential use of QSPT to differentiate the DLB constructional abilities from those of AD patients. The performance on QSPT of forty-six AD and forty-six matched DLB patients were then compared using univariate analysis of variance (ANOVA).

In order to verify the clinical value of QSPT in terms of differential diagnosis between AD and DLB, we compared linear vs non-linear analysis. The former consisted in a logistic regression on the qualitative dimensions of the QSPT, whereas the non-linear artificial neural networks methods were based on Twist system (Semeion).

Logistic regression on the qualitative dimension of the QSPT was performed using the SAS system. Twist system was used to build-up a predictive model based on the non linear combination of the items scores through advanced artificial neural networks. Twist system has been previously employed in different fields of predictive medicine with success by a number of groups [25–27].

Table 2
Univariate ANOVA of the scores provided by the two raters

| Parameters | Mean ± sd First rater | Mean ± sd Second rater | $F$ | p-value |
|---|---|---|---|---|
| Numbers of angles | 2.127 (0.169) | 2.456 (0.169) | 1.892 | 0.171 |
| Distance/Intersection | 2.246 (0.176) | 2.208 (0.76) | 0.24 | 0.878 |
| Closing/Opening | 1.281 (0.85) | 1.115 (0.85) | 1.884 | 0.172 |
| Rotation | 1.126 (0.91) | 1.111 (0.91) | 0.13 | 0.909 |
| Closing in | 0.955 (0.25) | 0.942 (0.25) | 0.136 | 0.713 |
| Total | 7.736 (0.464) | 7.832 (0.464) | 0.22 | 0.883 |

Table 3
Univariate ANOVA of intra-subjects scores

| Parameters | Mean ± sd Fist copy | Mean ± sd Second copy | $F$ | p-value |
|---|---|---|---|---|
| Numbers of angles | 3.45 (1.214) | 3.27 (1.421) | 0.104 | 0.750 |
| Distance/Intersection | 3.55 (1.214) | 3.73 (0.467) | 0.215 | 0.648 |
| Closure/Opening | 1.73 (0.647) | 1.64 (0.674) | 0.104 | 0.750 |
| Rotation | 1.64 (0.674) | 1.55 (0.688) | 0.098 | 0.757 |
| Closing-in | 0.91 (0.302) | 1.00 (0.100) | 1.000 | 0.329 |
| Total | 11.27 (3.875) | 11.18 (2.892) | 0.004 | 0.951 |

The TWIST system consists in an ensemble of two previously described systems: T&T and IS [27]. The T&T system is a robust data re-sampling technique that is able to arrange the source sample into sub-samples having similar probability density function. In this way, the data is split into two or more sub-samples in order to train, test and validate the ANN models more effectively. The IS system is an evolutionary wrapper system able to reduce the amount of data while conserving the largest amount of information available in the dataset. The combined action of these two systems allow us to solve two frequent problems in managing Artificial Neural Networks, i.e. the optimal splitting of the data set in training and testing subsets containing a balanced distribution of outliers and the optimal selection of variables with maximal amount of information relevant to the problem under investigation. Both systems are based on a Genetic Algorithm, the Genetic Doping Algorithm (GenD) developed at Semeion Research Centre [26]. A series of supervised Multi Layer Perceptrons, with four hidden units, were then used for the classification task. The final ANNs which were trained and tested on the new data set generated by TWIST system are "virgin" and operate independently and blindly from each other and from TWIST system. The classification results obtained from testing experiments allowed the calculation of the area under the curve (AUC) ROC and indexes of sensitivity and specificity.

The variables association scheme has been explored with Auto-Contractive map (Auto-CM). Auto-CM is a novel kind of unsupervised artificial neural network that makes possible to "spazialize" the associations between the variables inserted or adapted. This method is based on an artificial adaptive system able to define the association strength of each variable with all the others in any dataset, named the Auto Contractive Map (AutoCM). The architecture and mathematics of AutoCM is described elsewhere [28,29]. The Auto CM is a special kind of Artificial Neural Network developed at Semeion Research Centre, able to find, by a specific data mining learning algorithm, the consistent patterns and/or systematic relationships and hidden trends and associations among variables. The AutoCM connections matrix filtered by MST generates an interesting graph, whose biological evidence has been already tested in medical field [30,31].

## 3. Results

### 3.1. Inter-raters-reliability

The performances of each parameter (numbers of angles; distance/intersection between the two figures; closing/opening of the contour; rotation of one or both pentagons; closing-in) and the total score evaluated by the neuropsychologists (FD and SC) were entered into an univariate ANOVA. Results showed no statistical differences between the two raters (see Table 2), being Cronbach's $\alpha$ test respectively 0.88 and 0.97.

Table 4
Univariate ANOVA of DLB and AD patients (qualitative dimensions are reported in bold)

| Parameters | DLB Mean (SD) | AD Mean (SD) | p-value |
|---|---|---|---|
| MMSE Total | 19.39 (4.716) | 19.39 (4.529) | 1.000 |
| Temporal orientation | 2.54 (1.456) | 2.33 (1.492) | 0.481 |
| Spatial orientation | 4.02 (1.022) | 3.78 (1.114) | 0.286 |
| Immediate recall | 2.76 (0.603) | 2.76 (0.705) | 1.000 |
| Delayed recall | 0.91 (0.915) | 0.54 (0.912) | 0.055 |
| Attention | 2.50 (1.798) | 2.74 (1.666) | 0.510 |
| Naming | 1.98 (0.257) | 2.00 (0.000) | 0.568 |
| Repetition | 0.57 (0.501) | 0.98 (0.147) | 0.000* |
| Oral comprehension | 2.50 (0.723) | 2.50 (0.753) | 1.000 |
| Written comprehension | 0.83 (0.529) | 0.78 (0.417) | 0.663 |
| Written sentence | 0.70 (0.465) | 0.76 (0.431) | 0.487 |
| Constructional praxia | 0.22 (0.417) | 0.17 (0.383) | 0.604 |
| **Numbers of angles** | 1.74 (1.72) | 2.67 (1.41) | 0.005* |
| **Distance/Intersection** | 1.78 (1.66) | 2.50 (1.41) | 0.028* |
| **Closure/Opening** | 1.09 (0.94) | 1.52 (0.62) | 0.010* |
| **Rotation** | 1.22 (0.81) | 1.67 (0.56) | 0.002* |
| **Closing-in** | 0.91 (0.28) | 0.96 (0.21) | 0.404 |
| **Total qualitative score** | 6.74 (4.40) | 9.35 (3.41) | 0.002* |

*Significant differences between groups.

Table 5
Confusion matrix of ANNs modeling with predictive performances in two experiments (a-b and b-a sequence)

| ANN | Recs | LDB | AD | Sensitivity | Specificity | Overall accuracy | ROC AUC |
|---|---|---|---|---|---|---|---|
| FF_Sn 8 ab* | 46 | 21 | 25 | 80.95 | 72 | 76.48 | 0.71 |
| FF_Sn 8 ba** | 46 | 25 | 21 | 60 | 85.71 | 72.86 | 0.78 |
| Average | | | | 70.475 | 78.855 | 74.67 | 0.745 |

*Feed-forward Sine-net a-b sequence. **feed-forward Sine-net b-a sequence.

### 3.2. Intra-subjects reliability

A second univariate ANOVA comparing the performance obtained by the same patients in two separate session, showed no statistical differences (see Table 3).

### 3.3. DLB versus AD comparison

DLB performed worse than AD on number of angles, distance/intersection, closure/opening, rotation and total score, while no significant differences between the two groups were found on the closing-in phenomenon (see Table 4).

The findings obtained from this study demonstrated that the qualitative analysis of the pentagons' drawing seems to be a sensitive measure of visuo-constructive abilities in differentiating DLB from AD patients, suggesting the presence of different components of visuo-contructive abilities particularly vulnerable in DLB patients compared to AD.

Such results are in agreement with the described neuropsychological profile of DLB [32,33] where deficits on visuoperceptive and constructional abilities are more prominent than in AD. This type of deficits may be a sensitive indicator of DLB and might play a role in the differential diagnosis of this type of dementia [16]. However, even though significant at elicitating qualitative drawing profile of DLB compared to AD, the QSPT method could not add significant clinical value in terms of differential diagnosis.

A logistic regression on the same variables (SAS system) did not allow establishing a suitable modeling and a direct comparison between predictive results obtained.

### 3.4. Artificial neural networks

The application of TWIST system allowed the selection of a subgroup of 6 variables described in Table 3. This new data set has been analyzed with Back propagation ANNs employing a rigorous validation protocol. Twist system modeling showed a high predictive performance of the qualitative scoring method with fair sensitivity (70.29%) and good specificity (78.67%) (ROC-AUC 0.74), (See Table 5 and Fig. 1).

Auto-CM showed that the distribution of associations between demographical and cognitive variables and Lewy Body and Alzheimer's dementia, appeared

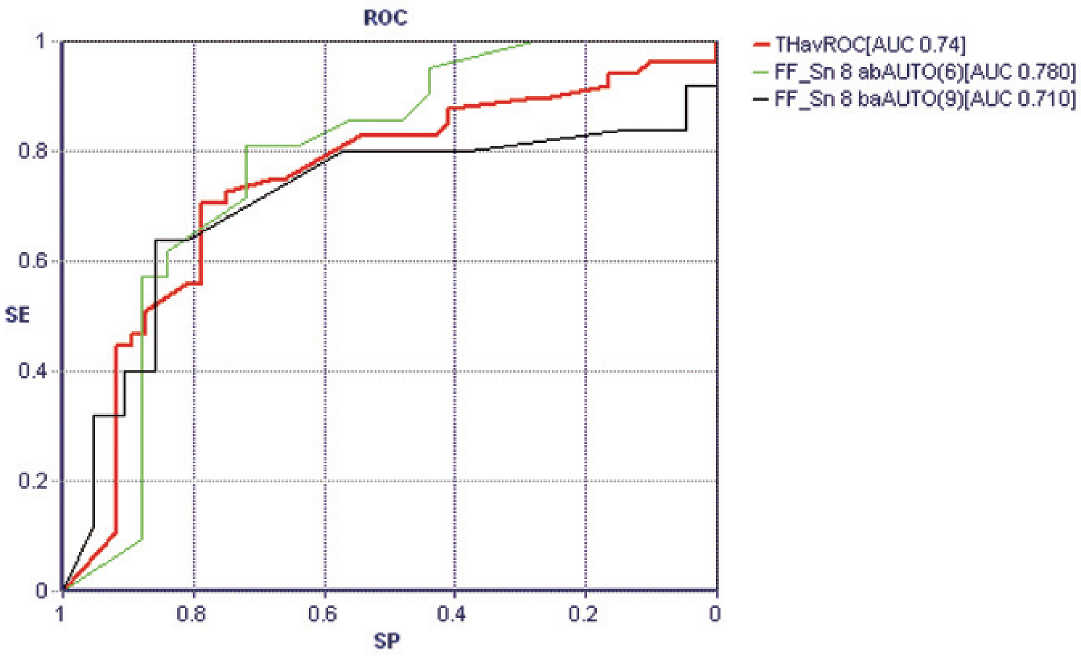

Fig. 1. Twist system modeling results (ROC-AUC 0.74).

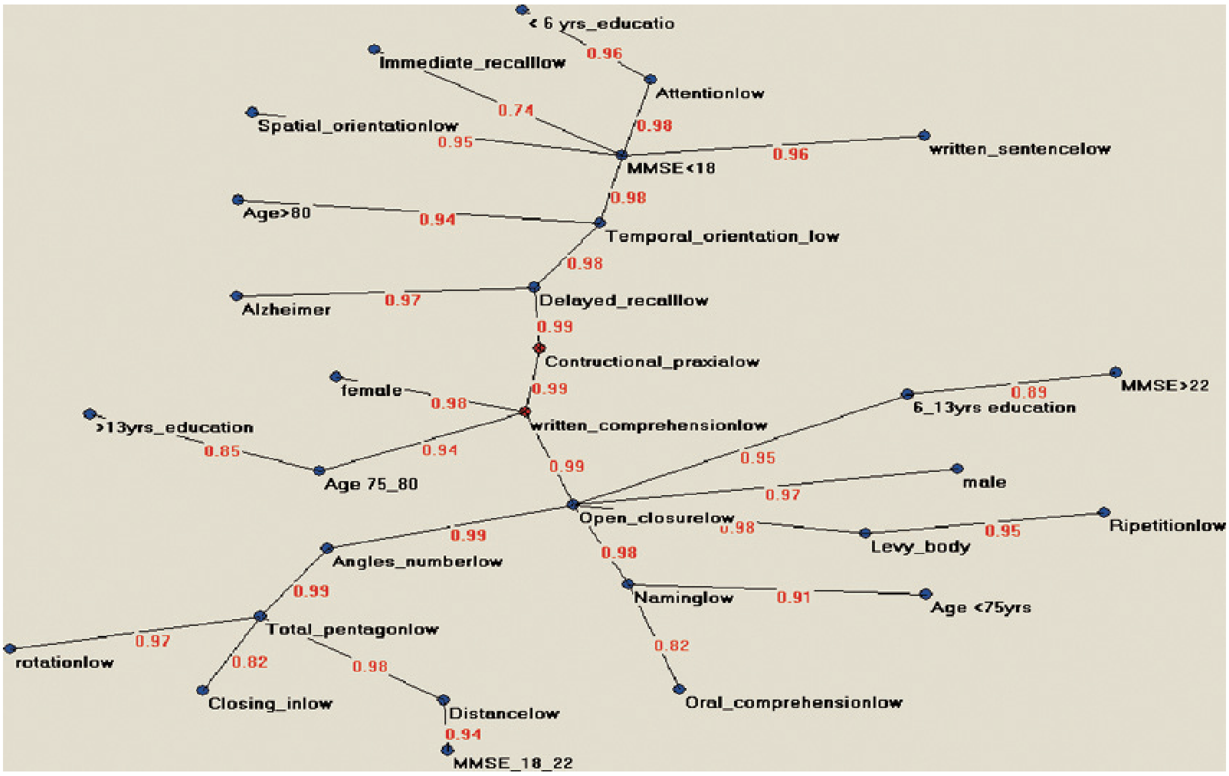

Fig. 2. Associations between demographical-cognitive variables and Lewy Body Dementia and Alzheimer’s dementia revealed by Auto-CM.

different. We considered as significant the degree of proximity across points which represent the strength of their reciprocal association, such as 1 and 2 points distance from the diagnostic label [25]. In particular, the diagnosis of Lewy Body was associated with low scores related to the opening/closure, repetition, number of angles, and to the language domain such as naming and written comprehension. Moreover, demographic variables of gender and education showed a relevant effect: male gender and middle high education, such as 6–13 years of education, were more associated with the DLB label. Alzheimer's dementia was mainly associated with delayed recall, temporal orientation and constructional apraxia (See Fig. 2).

This study showed that a qualitative approach of the MMSE pentagon test, not confined to a simple dichotomous scoring value, has a good inter-rater and intrasubject reliability. However the traditional statistical approach we first used by means of regression analysis was not able to support the clinical usefulness of QSPT in differentiating the visuo-perceptual deficits of DLB from those of AD patients, whereas the artificial neural networks gained better results.

Artificial neural networks showed that the qualitative parameters of pentagons copying represent a reliable and consistent measure of discrimination between DLB and AD. Even though the method of discriminating DLB from AD by visuospatial functions has been criticized [11] and few studies including MMSE pentagon copying analysis did not show any difference [7, 34], our results by non-linear analysis indicated that lower scores in some aspects of pentagons drawing, such as number of angles and opening/closure, could be more suggestive of a diagnosis of Lewy Body dementia, thus supporting other studies demonstrating better performance of AD in constructional tasks [16, 17].

However we can not omit that our study has a great limitation, due to lack of neuropathological verification and to possible presence of mixed pathology in DLB and AD. Even though this study provide a method to help clinicians to differentiate DLB from AD, the next step should focus on extending the observation to the autopsy verified patients, in order to definitely increase the clinical validity of the pentagons qualitative scoring approach.

## Acknowledgments

This study was supported by a grant from the Fondazione Cassa di Risparmio of Parma and Piacenza to PC and by funding from the Fondazione Bracco.
